# Reduced Graphene Oxide-Wrapped Super Dense Fe_3_O_4_ Nanoparticles with Enhanced Electromagnetic Wave Absorption Properties

**DOI:** 10.3390/nano9060845

**Published:** 2019-06-02

**Authors:** Qi Yu, Yiyi Wang, Ping Chen, Weicheng Nie, Hanlin Chen, Jun Zhou

**Affiliations:** 1 Faculty of Materials Science and Engineering & Liaoning Key Laboratory of Advanced Polymer Matrix Composites, Shenyang Aerospace University, Shenyang 110136, China; w13516017108@126.com (Y.W.); nie0301@126.com (W.N.); q13019399662@163.com (H.C.); toutes@163.com (J.Z.); 2 School of Chemical Engineering & State Key Laboratory of Fine Chemicals, Dalian University of Technology, Dalian 116024, China

**Keywords:** RGO/Fe_3_O_4_ nanocomposite, micromorphology, electromagnetic characteristics, microwave absorption properties

## Abstract

The efficient preparation of electromagnetic wave absorbing materials with low density and excellent electromagnetic wave absorption remains a considerable challenge. In this study, reduced graphene oxide (RGO) wrapped Fe_3_O_4_ nanoparticles (NPs) were synthesized based on one-step reaction by the reduction of graphene oxide (GO), and the generation of super-fine Fe_3_O_4_ NPs was achieved. The phase structure, chemical composition, micromorphology, and magnetism were characterized by X-ray diffraction (XRD), X-ray photoelectron spectroscope (XPS), scanning electron microscope (SEM), transmission electron microscope (TEM), and vibrating sample magnetometer (VSM), respectively. The electromagnetic characteristics were evaluated on a vector network analyzer by the coaxial line method. The results showed that super-fine Fe_3_O_4_ NPs with an average size of 6.18 nm are densely distributed on the surface of graphenes. The RGO/Fe_3_O_4_ nanocomposites exhibited excellent microwave absorption properties with a minimum reflection loss (RL) of up to −55.71 dB at 6.78 GHz at 3.5 mm thickness and the highest effective absorption bandwidth with RL values exceeding −10 dB is 4.76 GHz between 13.24 and 18 GHz at 1.7 mm thickness. This work provides a concise method for the development of RGO supported super dense Fe_3_O_4_ nanocomposites for high performance electromagnetic absorption applications.

## 1. Introduction

Due to the rapid development of electromagnetic wave detection technology, high performance microwave absorption materials have attracted more and more attention in the civil and military industries [1,2,3]. Ferrites have been widely used as electromagnetic wave (EW) absorbing agents due to their high saturation magnetization, low technological threshold, and cost [4,5]. Unfortunately, traditional ferrite absorbers have revealed shortcomings such as easy aggregation, high weight, and an inefficient EW absorption capability in practical applications. In general, these shortcomings are confined to the effects of magnetic loss when attenuating EW signals for Fe_3_O_4_ nanoparticles (NPs) due to their high resistivity, but a single loss mechanism is not beneficial for the achievement of ideal EW absorption performance. Thus, it is necessary to introduce some other types of electromagnetic loss mechanisms to alleviate this dilemma [6,7,8].

Recently, graphene nanosheets (GNs) have gained a lot of attention as microwave absorbers by virtue of their excellent conductivity and light weight [9,10,11]. Researchers have found that reduced graphene oxide (RGO) has attractive microwave absorbing ability owning to its high dielectric loss. Nevertheless, single RGOs could not achieve ideal microwave absorption performance due to their high permittivity and low permeability, which would lead to unfavorable electromagnetic impedance matching. In order to mitigate the dilemma, a lot of research has been conducted by combining RGO with magnetic components, such as FeCo [12], Ni [13], NiCoP [14], CoFe_2_O_4_ [15], and Fe_3_O_4_ [16]. For example, Xu et al. synthesized RGO/Ni hybrids with different mass ratios to obtain an optimal reflection loss value of −39.03 dB at 13 GHz [13]. Xue et al. synthesized NiCoP/RGO nanocomposites by one-pot reaction in order to improve dielectric and magnetic loss and thus enhance the reflection loss [14]. Chu et al. synthesized α-Fe_2_O_3_/RGO with a maximum reflection loss of up to −42.8 dB at a thickness of 1.8 mm [16]. Therefore, decorating magnetic metal NPs onto the large surface of GNs is a flexible strategy for improving microwave absorption properties by combining dielectric and magnetic loss mechanisms into a micro-nano composite structure, which can also improve their aggregation resistance and reduce their weight [17,18,19,20,21,22,23,24,25,26].

Herein, we report an easy and efficient method for the synthesis of graphene wrapped super dense Fe_3_O_4_ NPs via one-step reaction in order to enhance their microwave absorption properties. The phase structure, chemical composition, micromorphology, and magnetism of RGO/Fe_3_O_4_ nanocomposites are investigated, and the electromagnetic parameters and microwave absorption performance of RGO/Fe_3_O_4_ is evaluated.

## 2. Materials and Methods

All chemical reagents including ferric chloride (FeCl_3_), diethylene glycol (DEG), potassium permanganate (KMnO_4_), hydrogen peroxide (H_2_O_2_), concentrated sulfuric acid (H_2_SO_4_), and NaOH were purchased from Sinopharm Chemical Reagent Company (Shanghai, China). Graphite power was supplied by Yanhai Carbon Material Company (Qingdao, China). 

Graphene oxide (GO) was synthesized using modified Hummers method [27]. The source materials (2 g of graphene powder, 60 mL of concentrated H_2_SO_4,_ and 7 g of KMnO_4_) were successively put into a three-necked flask while undergoing mechanical stirring in an ice water bath. The mixture was heated to 35 °C while undergoing mechanical stirring for 3 h, and then diluted with distilled water (100 mL) dropwise. Afterwards, the mixture was heated to 90 °C while undergoing strong mechanical stirring for 30 min. Finally, distilled water (180 mL) and H_2_O_2_ (20 mL, 30%) were added dropwise and then the mixture was kept undisturbed for 24 h. The obtained precipitation was washed with HCl solution and distilled water through centrifugation until the decantate became neutral. Finally, the resulting graphene oxides (GOs) were obtained by ultrasonic treatment in water followed by freeze-drying.

The as-obtained GOs were firstly dissolved in 70 mL DEG, and 400 mg FeCl_3_ was added while the mixture was being stirred, then the suspension was heated to 220 °C while undergoing continuous stirring for 1 h with the protection of argon. Afterwards, NaOH solution was quickly poured into the suspension while undergoing stirring for another 0.5 h at 220 °C. Finally, the reaction system was cooled down to room temperature and the obtained RGO/Fe_3_O_4_ was separated and purified by centrifugation, washing, and drying. For comparison, pure Fe_3_O_4_ NPs was prepared using similar methods.

The chemical composition was characterized by X-ray photoelectron spectroscopy (XPS) performed on a Thermo ESCALAB 250 (Thermo Fisher Scientific Inc., Waltham, MA, USA) with Al-K_α_ radiation. The micromorphology was observed by transmission electron microscopy (TEM) conducted on a Tecna G2 F20 S-TWIN electron microscope (FEI Inc., Hillsborough, OR, USA) operated at 200 kV. The hysteresis loop was recorded on a SQUID-VSM vibrating sample magnetometer (Quantum Design Inc., San Diego, CA, USA). Electromagnetic parameters, including relative complex permittivity and permeability, were measured in the frequency range of 1–18 GHz using the coaxial line method on an AV3629D Vector Network Analyzer (CETI Co., Qingdao, China) by mixing the samples with paraffin wax (weight ratio of 1:1) and pressing them into a standard cylindrical shape mold with an inner diameter of 3 mm, an outer diameter of 7 mm, and a thickness of 3 mm.

## 3. Results and Discussion

### 3.1. Chemical Composition and Morphology

**Figure 1:**
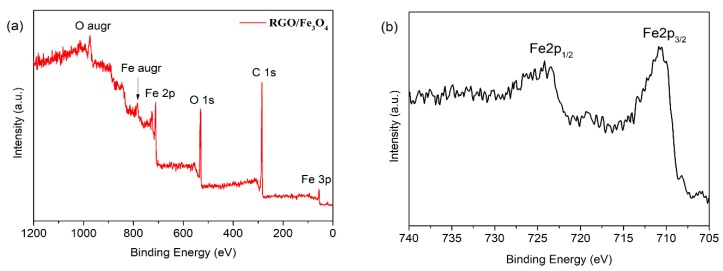
X-ray photoelectron spectroscope (XPS) spectra of reduced graphene oxide (RGO)/Fe_3_O_4_ nanocomposite: (**a**) full spectrum, (**b**) Fe2p high resolution spectrum.

The chemical composition of the RGO/Fe_3_O_4_ nanocomposite was identified by XPS as shown in Figure 1. Figure 1a shows the XPS full spectrum of RGO/Fe_3_O_4_. It can be observed that the peaks located at around 56, 285, 532, and 711.3 eV belong to Fe3p, C1s, O1s and Fe2p, respectively, which indicates that RGO/Fe_3_O_4_ consists of three major elements including C, O and Fe. In the Fe2p high resolution XPS spectra shown in Figure 1b, the peaks located at 711 and 723 eV are assigned to Fe 2p_3/2_ and Fe 2p_1/2_, respectively, which is consistent with the characteristic peaks of Fe_3_O_4_.

**Figure 2:**
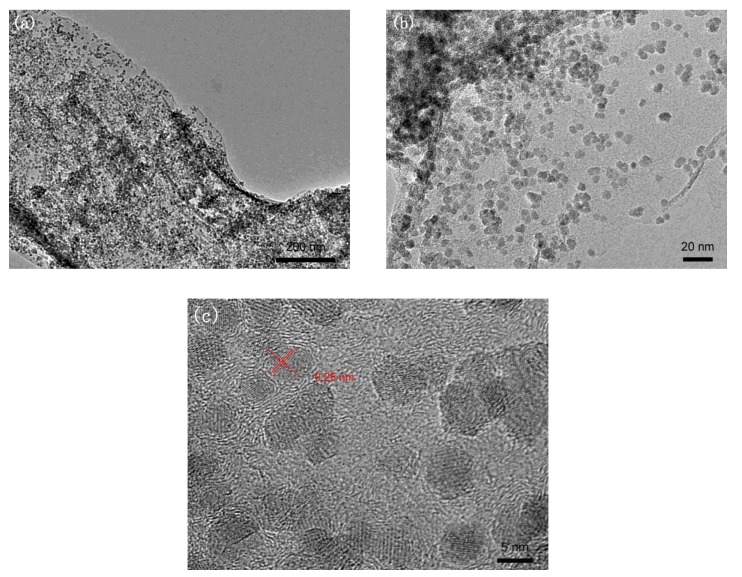
(**a**,**b**) TEM and (**c**) HRTEM images of RGO/Fe_3_O_4_ nanocomposite.

Figure 2 shows transmission electron microscopy (TEM) images of RGO/Fe_3_O_4_ nanocomposite. It can be seen from Figure 2a,b that the wrinkled surface of graphene nanosheets, which are capable of supplying a large loading area for NP growth, are homogeneously decorated with super dense spherical Fe_3_O_4_ NPs. The tiny Fe_3_O_4_ NPs, with an average size of 6.18 nm, are well distributed on the surface of the graphenes. In the loading process, GOs were employed as a flexible substrate for the in situ anchoring of Fe^3+^ and its growth into Fe_3_O_4_ NPs, so they played a confinement function to prevent the Fe_3_O_4_ NPs from detaching and aggregating. In the HRTEM image shown in Figure 2c, the interplanar distance of the NPs is 0.25 nm, which is in accordance with the lattice spacing of the (311) plane of cubic magnetite Fe_3_O_4_, further confirming the formation of Fe_3_O_4_ nanocrystals on the surface of RGO inferred from the XPS results.

### 3.2. Magnetic Properties

**Figure 3:**
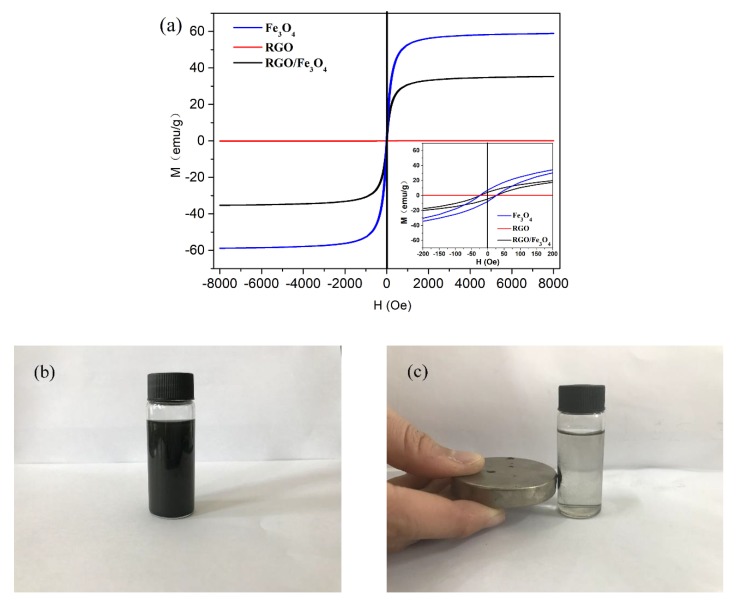
(**a**) Hysteresis loops of different samples measured at 298 K, (**b**) RGO/Fe_3_O_4_ nanocomposites dispersed in alcohol, and (**c**) separated by a magnet.

Figure 3a shows the hysteresis loops of different samples collected by a magnetometer at room temperature. The saturation magnetization (Ms) value of the RGO/Fe_3_O_4_, RGO, and Fe_3_O_4_ NPs are 36, 0.06, and 59 emu/g, and the corresponding coercivity (Hc) values are 25, 0, and 25 Oe, respectively. It can be observed that the Ms values for Fe_3_O_4_ NPs are higher than those of RGO/Fe_3_O_4_ and RGOs, indicating that the magnetism of RGO/Fe_3_O_4_ is introduced by loading magnetic Fe_3_O_4_ NPs onto the surface of nonmagnetic RGOs. Meanwhile, the Hc values of RGO/Fe_3_O_4_ and Fe_3_O_4_ NPs are the same, suggesting that the loading process has no effect on the intrinsic magnetic properties of Fe_3_O_4_ NPs. To further illustrate the magnetic properties, the RGO/Fe_3_O_4_ were dispersed in an ethanol solution (Figure 3b), which has favorable dispersibility and stability. After being attracted by a magnet (Figure 3c), the RGO/Fe_3_O_4_ dispersed in alcohol were quickly gathered together and attached to the bottle wall. Therefore, the graphenes were successfully magnetized by the loading of super dense Fe_3_O_4_ NPs. 

### 3.3. Electromagnetic Characteristics

In order to find out the essential reasons for microwave absorption mechanisms, the electromagnetic parameters, including the complex permittivity and permeability of Fe_3_O_4_ NPs, RGO, and RGO/Fe_3_O_4_ nanocomposites, were measured. The real parts (*ε*′ and *μ*′) symbolize the storage capacity of electric and magnetic energy, and the imaginary parts (*ε*″ and *μ*″) symbolize the energy loss, respectively. The dielectric loss (*tanδ_ε_* = *ε*″/*ε*′) and magnetic loss tangent (*tanδ_μ_* = *μ*″/*μ*′) give the balance between the real and imaginary parts in an absorbing structure. 

**Figure 4:**
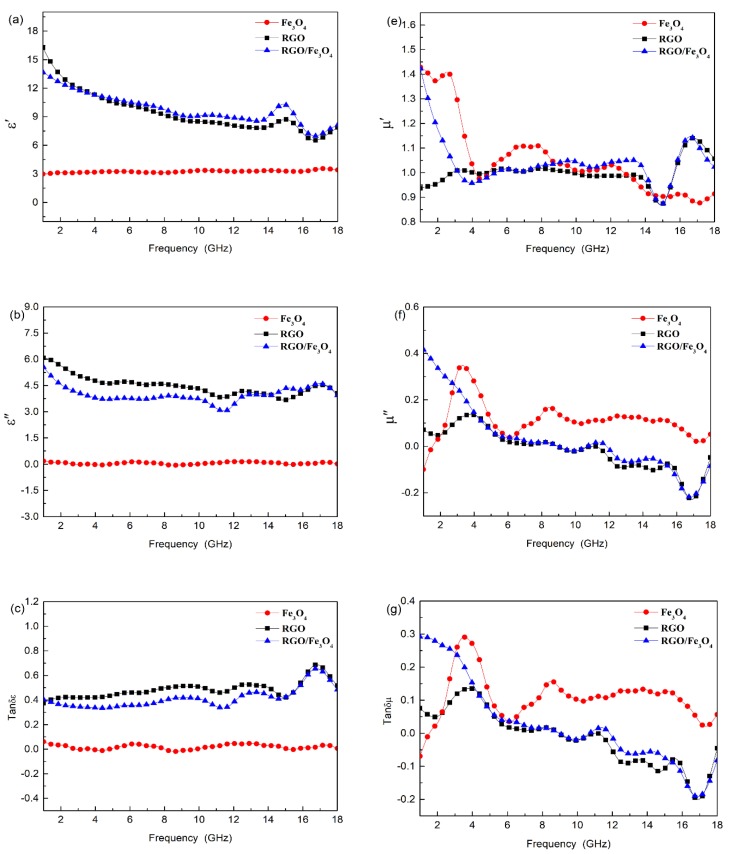

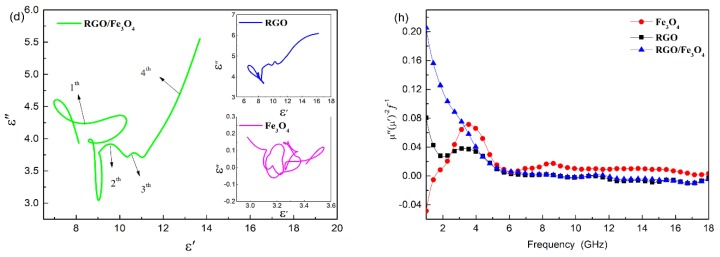
Electromagnetic characteristics of Fe_3_O_4_ nanoparticles (NPs), RGO and RGO/Fe_3_O_4_ nanocomposites: (**a**) real (ε′) and (**b**) imaginary (ε″) parts of complex permittivity; (**c**) dielectric loss tangent (*tanδ_ε_*); (**d**) Cole–Cole semicircles (*ε*″ vs. *ε*′); (**e**) real (*μ*′) and (**f**) imaginary (*μ*″) parts of complex permeability; (**g**) magnetic loss tangent (*tanδ_μ_*); and (**h**) *μ*″(*μ*′)^−2^*f*^−1^ vs. *f*.

Figure 4a–c show the frequency dependence of the real part (*ε*′) and the imaginary part (*ε*″) of complex permittivity, and the dielectric loss tangent (*tanδ_ε_*) for different samples. It is clear that the *ε*′, *ε*″, and *tanδ_ε_* values for both RGO/Fe_3_O_4_ and RGO are larger than those of Fe_3_O_4_ NPs. The *ε*′ of RGO/Fe_3_O_4_ declines from 13.69 to 6.98 with increasing frequency, and the *ε*″ remains relatively stable, changing from 5.55 to 3.04. The *tanδ_ε_* curve also exhibits a moderate growth trend ranging from 0.33 to 0.65 with some fluctuation, particularly in the high frequency region. Compared with pure RGOs, the RGO/Fe_3_O_4_ have a similar tendency in *ε*′, but it is slightly lower in *ε*″ and *tanδ_ε_*. The enhanced *ε*′, *ε*″, and *tanδ_ε_* of RGO/Fe_3_O_4_ is attributed to multiple dielectric loss behaviors derived from dielectric RGOs and magnetic Fe_3_O_4_ NPs. Firstly, the RGOs with high electric conductivity can form conducting networks, which is in favor of dielectric loss, thereby playing a main role in the substantial increase in *ε*′, *ε*″, and *tanδ_ε_* values. From the *ε*″ versus *ε*′ plot of the RGO/Fe_3_O_4_ (Figure 4d), it can be observed that there are multi-arcs for RGO/Fe_3_O_4_ and RGO, while there are no obvious arcs with increasing frequency for Fe_3_O_4_ NPs, indicating that debye dipolar relaxation is the main dielectric loss mechanism for RGO based nanostructure. In addition, the introduction of Fe_3_O_4_ NPs would create defects on the RGO surface, which would act as polarization centers for increasing dielectric loss. Secondly, although the sole Fe_3_O_4_ NPs with *ε*″ and *tanδ_ε_* approaching zero have hardly any dielectric loss, loading Fe_3_O_4_ NPs onto the surface of RGOs can introduce extra dielectric polarization behaviors. The interfacial polarization might be strengthened by a multi-interface between Fe_3_O_4_ NPs and graphenes, and the different electric potential between the two would induce charge accumulation at both ends, thus enhancing the space-charge polarization. The super-tiny Fe_3_O_4_ NPs have unsaturated bonds, which can serve as dipoles, thus the dipole polarization is enhanced [28]. The above mentioned polarization processes are beneficial for the improvement of dielectric loss and for the better dissipation of microwave energy.

Figure 4e–g show the real (*μ*′) and imaginary (*μ*″) parts of the relative complex permeability, and the magnetic loss (*tanδ_μ_*) for the different samples. It is seen that the *μ*′ values for RGO/Fe_3_O_4_ and Fe_3_O_4_ NPs sharply decrease initially and then become relatively stabilized with some fluctuation as the frequency increases. The *μ*″ and *tanδ_μ_* for Fe_3_O_4_ NPs have obvious resonance peaks at 2–6 GHz, while there is a decreasing trend with increasing frequency in the *μ*″ and *tanδ_μ_* curve for RGO/Fe_3_O_4_, which are favorable for enhancing magnetic loss at low frequencies [29]. The multiple resonance peaks are mainly attributed to natural resonance derived from magnetic Fe_3_O_4_ NPs. When the spherical Fe_3_O_4_ NPs are smaller, the anisortropy constant is higher, and the natural resonance is stronger. Meanwhile, exchange resonance may also contribute to magnetic loss by a small amount and to the anisotropy of magnetic NPs. In addition, the *μ*″(*μ*′)^−2^ƒ^−1^ values have obvious fluctuations at 1–6 GHz but remain relatively stable subsequently (Figure 4h), indicating that the eddy-current loss may come into action after 6 GHz.

### 3.4. Microwave Absorption Properties

**Figure 5:**
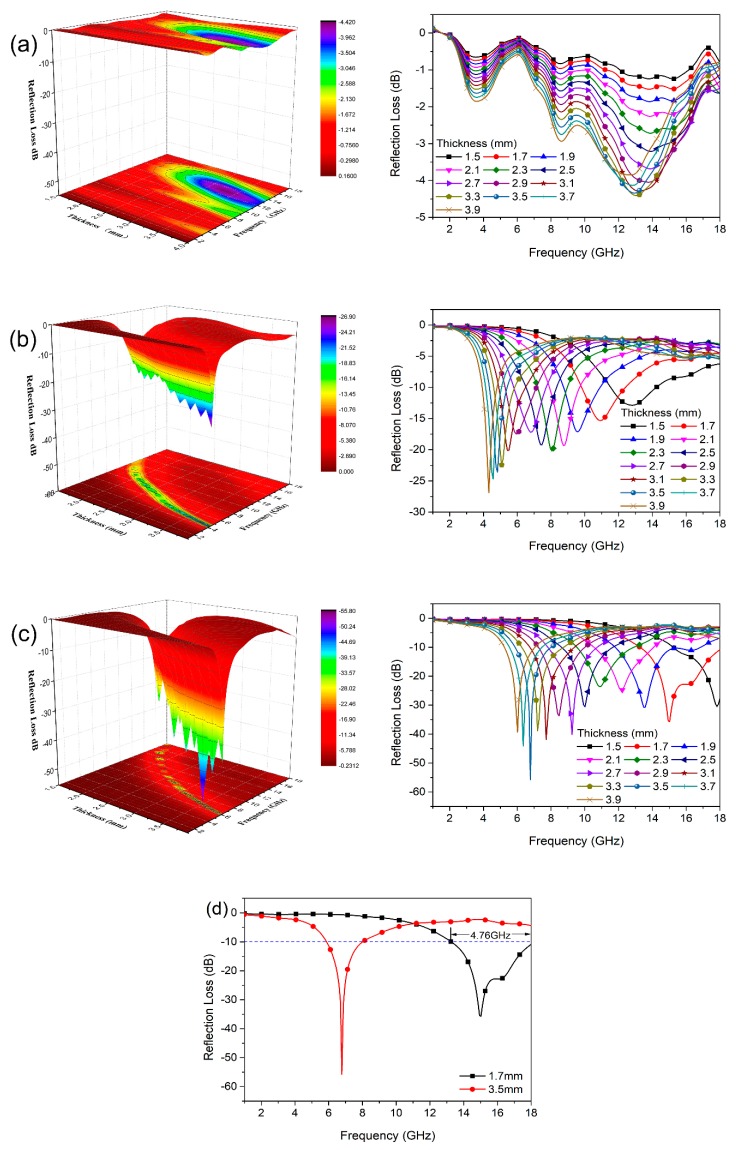
Reflection loss (RL) curves and 3D representation of (**a**) Fe_3_O_4_ NPs, (**b**) RGO, and (**c**) RGO/Fe_3_O_4_ with different thicknesses. (**d**) The RGO/Fe_3_O_4_ sample achieves an effective absorption bandwidth of 4.76 GHz at a thickness of 1.7 mm and reaches the maximum RL value of −55.71 dB (6.78 GHz) at a thickness of 3.5 mm.

Figure 5 displays the changes in reflection loss (RL) versus frequency for the samples at different thicknesses. Figure 5a shows that the absorption performance of Fe_3_O_4_ NPs is so poor that the minimum RL is merely −4.41 dB at 13.07 GHz at a thickness of 3.3 mm. For RGO, shown in Figure 5b, the absorption performance gets better, with the minimum RL increasing to −26.87 dB at a thickness of 3.9 mm and shifting to a lower frequency of 4.31 GHz. It is implied from Figure 5c that the incorporation of RGOs can shift the minimum RL of Fe_3_O_4_ NPs to a lower frequency region with enhanced microwave absorption and an enlarged effective bandwidth. The reflection loss of RGO/Fe_3_O_4_ nanocomposites is greatly enhanced, with the minimum RL value reaching up to −55.71 dB at 6.78 GHz with a thickness of 3.5 mm, and the highest effective absorption bandwidth with RL values lower than −10 dB is 4.76 GHz between 13.24 and 18 GHz at a thickness of 1.7 mm (Figure 5d). For comparison, the microwave absorption properties of dielectric/magnetic nanocomposites studied in similar works are displayed in Table 1.

**Figure 6:**
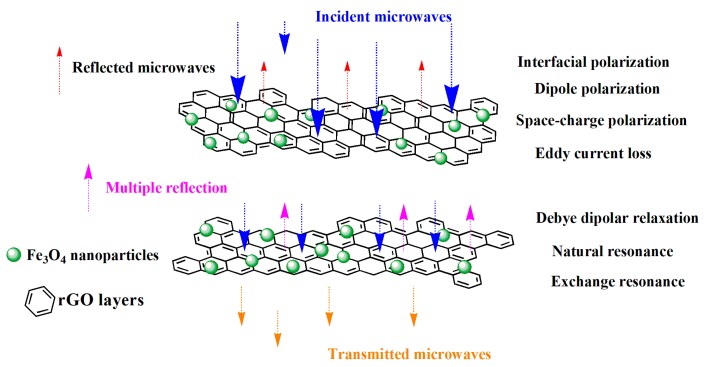
Diagram of microwave absorbing mechanisms for RGO/Fe_3_O_4_ nanocomposite.

Based on the above analysis, the enhanced microwave absorption properties of RGO/Fe_3_O_4_ nanocomposite can be attributed to multiple dielectric and magnetic loss mechanisms illustrated in Figure 6. The multi-interface introduced by super dense Fe_3_O_4_ NPs brought about extra polarization behaviors and magnetic loss, such as interfacial polarization, dipole polarization, space-charge polarization, eddy current loss, debye dipolar relaxation, natural resonance, and exchange resonance. All these processes improve the microwave absorption properties.

## 4. Conclusions

In summary, we have successfully synthesized RGO wrapped super dense Fe_3_O_4_ NPs via one-step reaction. The magnetic Fe_3_O_4_ NPs with an average size of 6.18 nm are well distributed on the surface of the graphenes. The RGO/Fe_3_O_4_ nanocomposites have shown excellent electromagnetic wave absorption properties. The minimum RL reaches up to −55.71 dB at 6.78 GHz at 3.5 mm thickness. The highest effective absorption bandwidth is 4.76 GHz between 13.24 and 18 GHz at 1.7 mm thickness. The multi-interface introduced by super dense Fe_3_O_4_ NPs brought about extra polarization behaviors and magnetic loss, both of which improved the microwave absorption properties. This work provides a concise way to develop graphene supported super dense Fe_3_O_4_ nanocomposites for high performance electromagnetic absorption applications.

## Figures and Tables

**Table 1:** Comparison of microwave absorption properties in this work and other representative works.

Absorber	Loading Ratio (wt%)	RL_min_ (dB)	Effective Bandwidth (GHz) (RL < −10dB)	Thickness (mm)	Refs
RGO/Ni	50	−39.03	4.3	2.0	[13]
RGO/NiCoP	50	−17.84	3.5	1.5	[14]
Fe_3_O_4_/GO/CNT	30	−37.3	2.2	5	[30]
G/BaFe_12_O_19_/CoFe_2_O_4_	50	−32.4	3.0		[31]
Fe_3_O_4_/CNT	50	−20.1	1.4	3.5	[32]
RGO/CoFe_2_O_4_	60	−39.0	4.7	2.0	[15]
RGO/matrimony vine-like Fe_3_O_4_	50	−42.8	4.6	1.8	[22]
RGO/Fe_3_O_4_	50	−55.71	4.76	1.7	This work
